# Intelligence

**Published:** 1831-07

**Authors:** 


### INTELLIGENCE.

### CHOLERA. REPORT OF THE PHYSICIANS.

The various documents relative to this malady which have been received by our government, have been transmitted to the College of Physicians. They were required to state their opinion of the mode in which the disease was propagated, and whether they deemed quarantine regulations necessary: the conclusion to which they have come is, that the disease is communicable by contagion from one person to another, and they do not consider it as certain that it may not also be transmitted by bales of goods or other merchandize; the propriety of strict quarantine is, of course, enforced. We have just heard that Dr. Barry, and Dr. Russel, of the India service, have been sent to Russia, for the purpose of making every inquiry respecting the disease. A third physician is also to be sent for the same purpose: we trust, therefore, that we shall speedily have more definite and substantial information upon the subject than has yet transpired.

### MADE AND ORDAINED BYE-LAWS OF THE ROYAL COLLEGE OF SURGEONS IN LONDON.

*By and at a Meeting of the Council of the said Royal College, holden at the College, on the 27th day of April,* 1831.

"We have examined and do approve of and allow these Bye-laws.

"Brougham, C.

"Tenterden,

"*21st day of May,* 1831." "N. C. Tindal."

No business whatever shall be transacted, nor any matter be discussed or debated at any meeting or assemblage convened by or under the authority of the President or Council, or before or after the business thereof shall have commenced, other than the particular business or matter in respect of which such meeting or assemblage shall have been convened; nor shall any debate or discussion whatsoever be had or allowed at any meeting convened by the President or Council for the delivery of lectures or orations, either before or after the same shall have commenced or terminated. And no meeting or assemblage of members of the College shall be held in the Hall or Council House of the College, or in any of its appurtenances, unless convened by or under the authority of the President or Council: and no member of the College shall advertise or convene or attend, or combine with others to advertise or convene or attend any meeting or assemblage in the Hall or Council House of the College, or in any of its appurtenances, not authorized by the President or Council. And any member of the College who may in any manner offend herein shall be liable to be restrained and excluded by the Council from attending any orations and lectures at the Theatre, and from any use of or admission to the Library and Museum, and to be suspended from any or all other privileges which he may have as a member of the College, for any such period as the Council may adjudge, or to removal by the Council from being a member of the College. And every member of the College who shall thereupon be removed as aforesaid shall forfeit all his rights and privileges as a member thereof.

All meetings convened by or under the authority of the President or Council of the College, as well for general business as for the delivery of orations or lectures, or for the distribution of prizes, shall be under the control and direction of the President or other member of the Council presiding at such meeting. And any member of the College who shall interrupt, impede, or interfere with the proceedings at any such meeting, or shall propose any matter for discussion or debate without the leave of the President or other person so presiding, shall, upon being required by the President or other person so presiding, immediately withdraw from such meeting; and shall be moreover liable to be restrained and excluded by the Council from attending any orations and lectures at the Theatre, and from any use of or admission to the Library and Museum, and to be suspended from any or all other privileges which he may have as a member of the College, for any such period as the Council may adjudge. And any member of the College who shall so offend a second time, or during any suspension by the Council shall attempt to exercise any of the privileges from which he shall be suspended, shall be liable to removal, by the Council, from being a member of the College. And every member of the College who shall thereupon be removed as aforesaid, shall forfeit all his rights and privileges as a member thereof.

---

### UNIVERSITY OF LONDON.

### *Annual Examinations for Prizes and Certificates of Honour.*

*May* 21*st*, 1831.

The Professor of Midwifery having submitted to the proprietors and friends of the institution an account of the progress and prospects of the University, at the conclusion of the third annual session, the prizes were awarded to the following successful candidates:

*Midwifery.* Gold medal: Mr. Peter Martin, of Reigate, Surrey. First silver medal: Mr. Thomas Howitt, of Lancaster. Second ditto: Mr. Peter Hulme Edge, of Salford. Eleven certificates of honour.

*Anatomy.* Gold medal: Mr. James Long, of Camden street, Camden Town. First silver medal: Mr. Joseph Thompson, of Colston Basset, Notts. Second ditto: Mr. Richard Wakefield, Judd place, New Road. Nineteen certificates of honour.

*General Anatomy.* Gold medal: Mr. Robert Grueber Shute, of London. First silver medal: Mr. John Storrar, of London. Second ditto: Mr. James Long, of Camden Town. Twenty-three certificates of honour.

*Nature and Treatment of Diseases.* Gold medal: Mr. Thomas Eden, of Liverpool. First silver medal: Mr. Robert Docksey Goodwin, of Ashbourne. Second ditto: Mr. Peter Hulme Edge, of Salford. Eighteen certificates of honour.

*Materia Medica.* Gold medal: Mr. David William Nash, of Bristol. First silver medal: Mr. J. N. Huddleston, of London. Second ditto: Mr. Frederick Edmonds, of Penzance. Fifteen certificates of honour.

*Surgery.* Gold medal: Mr. James Long, of Camden street, Camden Town. First silver medal: Mr. James Thompsom, of Colston Basset, Notts. Second ditto: Mr. D. W. Nash, of Bristol. Twenty-five certificates of honour.

*Chemistry.* Gold medal: Mr. D. W. Nash, of Bristol. First silver medal: Mr. Collings Mauger Carre, of Guernsey. Second ditto: Mr. Henry Cook, of London. Thirty-two certificates of honour.

*Physiology.* Gold medal: Mr. Henry Plank, of London. First silver medal: Mr. James Wearne, of St. Ives, Cornwall. Second ditto: Mr. Peter Martin, of Reigate. One certificate of honour.

*Comparative Anatomy.* Gold medal: Mr. Charles Le Mann. Silver medal: Mr. Robert Garner, of the Potteries, Staffordshire. Five certificates of honour.

*Botany.* One prize and four certificates.

The situation of assistant-surgeon in the service of the East-India Company, which had been placed at the disposal of the Council by the Right Hon. Charles Wynne, subject, however, to the obligation of conferring it upon the most successful student of his year, who might choose to become a candidate for the nomination, was awarded to Mr. Evans, after a very severe public examination, in which he highly distinguished himself. The chairman, Sir Thomas Denman, complimented the students generally upon the very favorable report that had been given by the different professors, of their zeal in the prosecution of their studies, and of the great and decided progress which they had made in the various branches of medical science. He also alluded to the flattering testimony which had been borne to the conduct and talent of Mr. Evans; and he observed, that the annals of the University, in recording the memorials of her distinguished pupils, would bear his name as the first who had had the resolution to present himself before a public meeting, to undergo a searching examination by the whole of the medical professors.

It would have been gratifying to us to give a brief account of the examinations in each of the different classes, but we can only select a part of one, that for Materia Medica and Therapeutics. The questions proposed by the professor, Dr. A. T. Thomson, were thirty-five in number; and the answers given by the gentleman who obtained the gold medal evince an extent of information, that reflects the highest credit upon his talent and industry.

The following are a part of the questions proposed by Dr. Thomson, with the answers given by Mr. David William Nash.

QUESTIONS.

"20. A man is labouring under Hæmoptysis, and is taking Acetate of Lead to subdue the hæmorrhage; and, at the same time, a mixture containing Acetate of Ammonia: he is attacked with Colica pictonum: what is the cause of this? and how is such an inconvenience to be avoided, without setting aside the use of either medicine?

"21. In very irritable states of the stomach, which prevent Sulphate of Quinia from being administered in the usual manner, how should it be exhibited?

"22. What states of the habit, and what external circumstances impede and favor the operation of Diuretics?

"23. A large dose of Opium administered immediately after bleeding, in inflammatory complaints, often acts so beneficially as to render the repetition of the bloodletting unnecessary: what is the theory of this effect of Opium?

"24. What is the cause of the pale alvine evacuations which frequently attend the administration of Opium?

"25. Describe the diagnostic symptoms which indicate poisoning by Opium; and state the chemical proofs which demonstrate that Opium was the poison employed?

"28. What are the most obvious effects of a large dose of Acetate of Morphia?

"27. Under what states of the habit does Opium augment the activity of purgatives?

"28. When diluted Nitric Acid is distilled on Balsam of Peru, Hydrocyanic Acid is contained in the product: explain the theory of this result?

"29. Independent of odour, how is the presence of Hydrocyanic Acid detected in mixed fluids?

"30. What are the effects of this Acid on the system?

"31. What is the theory of its operation in relieving Dyspepsia?

"32. Explain the difference in the action of Sedatives and Narcotics, of Tonics and Astringents, in reference to the Nervous System: what is the most probable theory explanatory of the operation of each of these orders of medicines on the living system?

"33. How do Mercurials operate in relieving Amenorrhœa?

"34. In what manner is Electricity to be applied for relieving Habitual Costiveness, without the use of purgatives?"

ANSWERS.

"20. If the solution of Acetate of Ammonia were perfectly free from the presence of carbonic acid, Acetate of Lead might be prescribed with it with safety; but as, from the mode of its preparation, this is seldom the case, the acetate of lead is decomposed, a carbonate of lead is formed, and symptoms of Colica pictonum supervene. This accident may be prevented from recurring, by directing the solution of acetate of ammonia to be warmed previously to its administration, in order that any carbonic acid which it contains may be expelled.

"21. If the stomach were so irritable as not to bear the Sulphate of Quinia, this medicine may be rubbed upon the gums and on the inside of the cheeks of the patient, when its beneficial effects will be produced upon the system in the same manner as if taken into the stomach.

"22. In prescribing diuretics, it is necessary to take care that no inflammatory symptoms are present; that the surface be kept cool; or, the exhalants of the skin being stimulated by warmth, the effects of the medicine will be determined to the skin, and diaphoresis produced: diarrhœa is equally to be avoided. The daytime should be chosen for their exhibition, as exercise promotes their efficacy; diluents should be freely supplied, and the patient restricted from the use of stimulant food.

"23. When a large dose of Opium is given immediately after bleeding, the stimulant effects of the medicine are so trifling as to be almost imperceptible, and the sedative influence comes immediately into view, diminishing arterial action, and allaying that susceptibility to morbid impressions which must be overcome before health can be restored.

"24. It is found that, during the administration of opium, the evacuation becomes pale; an effect to be ascribed to a deficiency of bile, consequent on a paralytic state of the gall-ducts, which is produced by opium.

"25. When opium has been taken in a dose sufficiently large to prove fatal, giddiness and stupor generally come on without much, if any, previous excitement. Very soon the stupor increases, and symptoms resembling apoplexy supervene; the patient lies insensible to external impressions; the breathing slow, the eyes half open, the pupils very much contracted; the pulse almost imperceptible, and the whole expression of the countenance that of deep and perfect repose. As the poisoning advances, the features assume a ghastly appearance; the muscles become relaxed; the respiration almost ceases; the stupor passes into pure coma, and the patient expires. Sometimes convulsions precede death. The chief diagnostic symptoms are the possibility of rousing the patient to consciousness, when in the deepest lethargy, by forcibly shaking him, or speaking in a loud voice, or injecting water into his ear.

"To ascertain the presence of opium, procure a fluid solution by boiling the contents of the stomach, and the vomited matters, in a sufficient quantity of distilled water, acidulated with acetic acid; filter, and add a solution of acetate of lead as long as any precipitate falls: this precipitate is meconate of lead; the fluid contains acetate of morphia.

"Take the meconate of lead, diffuse it in water, and transmit a stream of sulphuretted hydrogen gas through the fluid; filter, and then boil to expel any excess of sulphuretted hydrogen, and filter again through animal charcoal. The solution of meconic acid may now be tested.

"The solution of permuriate of iron will strike an intense cherry-red colour: no other acid, except the sulphocyanic, will produce a similar colour, and this may be distinguished when compared with a test previously prepared from solid opium, or, according to Dr. Clendinning, by the addition of a small quantity of a solution of pure potassa, which destroys the colour of the sulphocyanate, but renders that produced by the meconate more intense. Sulphate of copper gives an emerald-green colour, (similar to that thrown down from an arsenical solution by the same reagent,) which soon becomes yellow. The solution of acetate of morphia, which was left, being deprived of its acetic acid by a little ammonia, the morphia will strike an orange red with nitric acid, and an indigo blue with the solution of permuriate of iron.

"26. When a large dose of Acetate of Morphia has been taken, there is hardly any perceptible excitement, but insensibility, peculiar tremors, and a clammy perspiration on the lower parts of the body, and an intense itching of the skin, are the most obvious effects.

"27. In spasmodic affections, as Ileus and Colica pictonum, Opium allays the irritability, and relieves the spasmodic contractions of the intestines, enabling the purgative to exert its proper influence in accelerating the peristaltic movements of the intestinal canal.

"28. Balsam of Peru, like many other vegetable products, is composed, as to its ultimate elements, of oxygen, hydrogen, and carbon; and during the distillation of the balsam with nitric acid, a new arrangement of these elements is produced: the carbon of the balsam, and the nitrogen of the acid, combine to form bicarbonate of nitrogen, or cyanogen. This again unites with hydrogen, to form hydrocyanic acid.

"29. In testing for Hydrocyanic Acid in mixed fluids, we take advantage of its volatility, and having diluted the mixture with distilled water, and acidulated with acetic acid, to neutralize any alkali by which the hydrocyanic acid may be retained, the fluid is to be placed in a water bath, and the acid distilled over into a receiver surrounded by ice. It is to be tested by,

"1. Add a drop of a solution of potassa, and then a similar quantity of a solution of the protosulphate of copper, and then a little dilute sulphuric acid: if hydrocyanic acid be present in an extremely minute quantity, a milkiness will become evident.

"2. Treat it in the same way, substituting the protosulphate of iron for the protosulphate of copper, when a blue precipitate will be formed, gradually deepening on exposure to the air, till it acquires the characteristic tint of Prussian blue.

"30. Hydrocyanic Acid operates as a direct sedative on the nervous energy, at once destroying sensation and consciousness to external impressions. Operating immediately on the nerves of sensation, the animal functions are not primarily affected, which accounts for the fact that, after the sensitive life of an animal poisoned by this acid has ceased, the functions of respiration, the action of the heart, and the peristaltic movement of the intestines still continue for some length of time.

"31. In Dyspepsia there is always subacute inflammation, and a morbid irritability of the mucous membrane of the stomach. Hydrocyanic Acid allays this irritability, and permits a slow and healthy secretion of the gastric juice.

"32. Sedatives directly diminish the susceptibility to impressions, and destroy the vital energies without producing previous excitement. Narcotics produce a temporary excitement, increase the susceptibility to impressions, rouse the powers of the system for a time, and are succeeded by a collapse which is more than proportional to the previous excitement. Tonics stimulate the nervous energy, but in so slight a degree that their administration requires to be continued for some time before their effects on the system are rendered visible; and they are not followed by a collapse. Astringents operate directly on the nerves of motion corrugating and condensing the muscular fibres of the part to which they are applied; their action on the nerves of sensation being secondary, and the sensation of astringency which they produce is caused by the compression of the ultimate extremities of the sentient nerves, consequent on the contraction of the muscular fibre. They do not increase the sensibility of the parts to which they are applied.

"33. Mercurials exert a specific influence on the whole glandular system, and consequently stimulate the uterus to a more due and healthy performance of its natural functions. Amenorrhœa depends on a diminished action in the vessels of the uterus, which are rendered unable to exercise the powers of secretion with which they were endowed when in a state of health. Mercury produces an increased action in the whole secerning system, and restores the debilitated vessels of the uterus to the exercise of their natural powers.

"34. In applying Electricity, we must recollect that this is a very powerful stimulant, and must first begin with the application of the electric aura to the abdomen of the patient; and when the stimulus, from repetition, fails to produce the desired effect, sparks taken from the surface of the abdomen, by insulating the patient, and applying a conductor to the surface, must be resorted to."

---

DINNER IN HONOUR OF JOSHUA BROOKES, ESQ. F.R.S. F.L.S. &c.

**MANY of the former pupils of this highly distinguished anatomist invited him to a public dinner, on the 25th June, at the Freemason's Tavern. The chair was occupied by Dr. BREE. In the course of the evening, many of the gentlemen present took the opportunity of expressing their esteem and respect for the talents and private worth of their late celebrated teacher. We ourselves had the benefit of Mr. BROOKES' instruction in anatomy, and it is very gratifying to us to bear our testimony to the uniform kindness and unceasing attention with which he watched and directed the progress of his pupils in their anatomical and surgical studies.**

---

### BIBLIOGRAPHICAL NOTICE.

### METEOROLOGICAL JOURNAL,

By Messrs. Harris and Co. Mathematical Instrument Makers, 50, High Holborn.

| | Moon. | Rain gauge. | Thermom. | | | Barometer. | | De Luc's Hygrometer. | | Winds. | | Atmospheric Variation. | | |
|---|---|---|---|---|---|---|---|---|---|---|---|---|---|---|
| May | | | 9 a.m. | max. | min. | 9 a.m. | 10 p.m. | 9 a.m. | 10 p.m. | 9 a.m. | 10 p.m. | 9 a.m. | 2 p.m. | 10 p.m. |
| 20 | | .45 | 61 | 65 | 55 | 29.63 | 29.68 | 48 | 49 | SSW | SSW | Cloudy | Fine | Cloudy |
| 21 | | | 58 | 62 | 48 | .72 | .80 | 51 | 54 | NNE | N | Fine | — | Fine |
| 22 | | | 56 | 67 | 53 | .76 | .68 | 55 | 57 | NW | | — | — | — |
| 23 | | | 62 | 70 | 55 | .61 | .67 | 58 | 59 | | NE | Cloudy | — | — |
| 24 | | | 63 | 65 | 56 | .62 | .62 | 62 | 59 | E | E | Overc. | — | — |
| 25 | | | 60 | 67 | 53 | .64 | .68 | 53 | 52 | ENE | SE | Fine | — | — |
| 26 | ○ | .22 | 62 | 63 | 51 | .64 | .62 | 52 | 53 | ENE | ESE | — | — | — |
| 27 | | | 55 | 64 | 52 | .60 | .62 | 56 | 56 | NE | ENE | — | — | — |
| 28 | | | 60 | 61 | 48 | .73 | .82 | 56 | 57 | ENE | SSW | — | — | — |
| 29 | | | 51 | 56 | 50 | .78 | .74 | 59 | 69 | E | E | Rain | Rain | Sleet |
| 30 | | 1.45 | 56 | 62 | 53 | .70 | .76 | 75 | 68 | NE | SE | Cloudy | Fine | Fine |
| June | | | | | | | | | | | | | | |
| 1 | | | 62 | 65 | 51 | .79 | .72 | 60 | 60 | SE | E | Fine | — | Overc. |
| 2 | | | 57 | 62 | 50 | .75 | .84 | 54 | 51 | ENE | ENE | — | — | Fine |
| 3 | ☾ | | 62 | 70 | 52 | .92 | .93 | 48 | 48 | NE | SE | — | — | — |
| 4 | | | 62 | 62 | 51 | .97 | .97 | 48 | 47 | ESE | ENE | — | — | — |
| 5 | | | 61 | 64 | 53 | 30.00 | .98 | 47 | 45 | NNE | SE | — | — | — |
| 6 | | | 61 | 66 | 51 | 29.91 | .84 | 47 | 50 | E | | — | — | Cloudy |
| 7 | | | 60 | 60 | 57 | .87 | .90 | 48 | 48 | N | NE | Cloudy | — | — |
| 8 | | | 58 | 60 | 52 | .86 | .73 | 48 | 49 | NNW | W | Fine | — | Fine |
| 9 | | | 60 | 67 | 53 | .63 | .62 | 49 | 49 | W | E | Cloudy | — | — |
| 10 | ● | | 67 | 69 | 58 | .64 | .61 | 49 | 48 | W | SSW | Fine | — | Rain |
| 11 | | .86 | 68 | 68 | 58 | .54 | .56 | 48 | 47 | W *va.* | SW | — | Rain | Cloudy |
| 12 | | | 68 | 69 | 55 | .40 | .60 | 47 | 46 | W *va.* | WSW | Cloudy | Fine | Sho'ry |
| 13 | | .25 | 62 | 68 | 60 | .63 | .72 | 48 | 51 | W | SW | Fine | — | Cloudy |
| 14 | | | 67 | 69 | 59 | .69 | .80 | 54 | 53 | WNW | WNW | Cloudy | Rain | Fine |
| 15 | | | 68 | 72 | 58 | .95 | .87 | 50 | 49 | W | SW | Fine | Fine | — |
| 16 | | .29 | 61 | 68 | 58 | .69 | .65 | 47 | 49 | SW | SSW | Fine | — | — |
| 17 | ☽ | | 65 | 66 | 57 | .65 | .64 | 49 | 49 | WSW | SW | Cloudy | — | Fine |
| 18 | | | 66 | 68 | 58 | .74 | .79 | 49 | 51 | SW | SW | Cloudy | — | — |
| 19 | | | 68 | 70 | 52 | .82 | .76 | 51 | 52 | SW | S | Cloudy | — | Cloudy |

The quantity of Rain fallen, in the month of May, was 2 inches and 12 100ths.

